# Isolation of Staphylococcus aureus Urinary Tract Infections at a Community-Based Healthcare Center in Riyadh

**DOI:** 10.7759/cureus.35140

**Published:** 2023-02-18

**Authors:** Mohammad K Alshomrani, Ahmad A Alharbi, Abdullah A Alshehri, Muhammad Arshad, Saeed Dolgum

**Affiliations:** 1 Microbiology, Riyadh Regional Laboratory and Blood Bank, Riyadh, SAU; 2 Pathology, Majmaah University, Al Majma'ah, SAU; 3 Laboratory, Prince Mohammed Bin Abdulaziz Hospital, Riyadh, SAU; 4 Laboratory, Dr. Sulaiman Al-Habib Hospital in Al Takhassusi, Riyadh, SAU; 5 Pediatric Infectious Diseases, Dr. Sulaiman Al-Habib Hospital in Al Takhassusi, Riyadh, SAU

**Keywords:** antibiotic resistance, urine culture, urinary tract infection, bacteriuria, staphylococcus aureus

## Abstract

Background

The aim of this study was to assess the clinical presentation, risk factors, and comorbidities of the patients with *Staphylococcus aureus* bacteriuria, and to analyze the antimicrobial susceptibility data of *S. aureus* isolated from their urine samples.

Methods

A total of 90 isolates of *S. aureus* were collected from patients with urinary tract infections (UTIs). Urinalysis was performed manually, including macroscopic examination of color and appearance, and microscopic examination for the presence of urinary WBCs, RBCs, and bacteria. Full identification and susceptibility testing of *S. aureus* were performed by the VITEK 2 system (BioMérieux, Marcy-l'Étoile, France) using standard criteria.

Results

The majority of the patients were female (62%), with a mean age of 32.9 years. Most of the patients were outpatients (85%), and 52% were previously healthy with no underlying disease. Seventy positive urine cultures were associated with UTI symptoms, and the most common symptom was dysuria (40%). Out of 77 urinalyses performed, 58 were positive for UTI. Of the *S. aureus* isolated, 24% were methicillin-resistant *S. aureus* (MRSA). Susceptibility to vancomycin, teicoplanin, and linezolid was 100%, while susceptibility to erythromycin, clindamycin, gentamicin, trimethoprim-sulfamethoxazole, fusidic acid, and tetracycline, was 86%, 93%, 97%, 91%, 68%, and 87%, respectively.

Conclusion

Although *S. aureus* UTI is known to be associated with other risk factors such as urinary catheterization, long hospital stay, or complicated UTI, our results show the community-acquired presentation of UTI. Trimethoprim-sulfamethoxazole may be used as an effective treatment for UTI caused by *S. aureus*. *S. aureus* UTI could be an alarming sign of more invasive infections such as *S. aureus* bacteremia, though clinical evaluation and finding the source of *S. aureus* are crucial for effective treatment and prevention of further complications.

## Introduction

*Staphylococcus aureus* is a notorious human pathogen that causes a wide range of clinical infections. It is a major cause of bloodstream infections and infective endocarditis as well as osteoarticular, skin and soft tissue, pleuropulmonary, and implant-associated infections [1]. Although *S. aureus* accounts for only 0.5-6% of urinary tract infections (UTIs), untreated infection can lead to severe life-threatening conditions [2]. Isolation of *S. aureus* from urine samples must be investigated further to rule out staphylococcal bacteremia arising from elsewhere (e.g., in cases of endocarditis). Nonetheless, in certain patients, *S. aureus* causes ascending urinary tract colonization and infection. This usually occurs in patients with urinary tract instrumentation and/or the presence of an indwelling catheter [3,4]. Although the majority of *S. aureus* bacteriuria cases are asymptomatic, when symptoms appear, the most common symptom of *S. aureus* UTI is fever. Other symptoms include hematuria, altered mental status, dysuria, suprapubic pain, and, less commonly, flank pain [5]. Methicillin-resistant *S. aureus* (MRSA) UTI is associated with recent antibiotic use and urinary catheterization and leads to longer stays in healthcare facilities. The strong association of catheters with UTI signifies the importance of reducing the use of urinary catheterization to essential cases only and removal of the device as soon as clinically indicated. Patients with catheter-associated *S. aureus* UTI, after excluding bacteremia, should be treated for 10 to 14 days with appropriate antimicrobials, as determined by culture and susceptibility results, as well as removal or replacement of the catheter [1].

Several studies reported *S. aureus* UTI in skilled-care nursing homes, long-term care facilities, and attending specialist and tertiary hospitals [3,4,6,7]. The aim of this study was to assess the clinical presentation, risk factors, and comorbidities of the patients with *S. aureus* bacteriuria, and to analyze antimicrobial susceptibility data of *S. aureus* isolates from urine samples in a community-based healthcare center.

## Materials and methods

Study population and sample collection

A retrospective observational cohort study was conducted at Dr. Sulaiman Al-Habib Hospital (HMG), Takhassusi branch in Riyadh, Saudi Arabia, and approved by the Institutional Review Board (IRB) of HMG (approval number: RC20.12.117). A total of 85 patients with *S. aureus* bacteriuria (positive urine culture for *S. aureus*) were enrolled in this study from the first of January, 2017 to the end of June, 2020. The initial *S. aureus* isolated from urine culture was included for all the study subjects. If the subsequent *S. aureus* from urine culture for the same patient showed identical susceptibility results, they were considered as a single isolate, and the second one was excluded. However, if the second isolate had a different susceptibility result, it was considered a different strain and included in the total number of *S. aureus* isolates (n = 90). Four patients had more than one isolate with different susceptibility results, and all were included. Registries containing patients' demographics and clinical and laboratory data were extracted from the VIDA system (HMG hospital information system).

Urinalysis

Urinalysis was performed manually, including the macroscopic examination (color and appearance) and microscopic examination for the presence of urinary WBCs, RBCs, and bacteria. A chemistry analyzer (CLINITEK Advantus, Siemens Healthineers, Erlangen, Germany) was used to measure nitrates and leukocyte esterase.

Urine culture

The culture results were interpreted based on the colony count, the number of organisms, and the presence of symptoms [8]. Full identification and susceptibility testing of *S. aureus* were performed by the VITEK 2 system (BioMérieux, Marcy-l'Étoile, France) using the manufacturer’s recommendations. The VITEK system was used to detect MRSA by cefoxitin screening and performing oxacillin minimum inhibitory concentration.

Statistical analysis

Frequency and percentages were used for descriptive analysis, and cross-tabulation was used to identify the relationship between categorical variables. Using the chi-square test, a p-value of 0.05 or less was considered significant. All data analysis was performed with SPSS version 26 (IBM Corp., Armonk, NY).

## Results

From January 2017 to June 2020, we identified and included 85 consecutive patients in the study. A total of 81 *S. aureus* were isolated from 81 patients, while three *S. aureus* were isolated from one patient, and two *S. aureus* were isolated from each of the three patients. The majority of the patients were female (62%) and the ages ranged from less than one month to 83 years with a mean age of 32.9 years. Most of the patients (85%) were outpatients. Of the patients, 47 were previously healthy with no underlying disease while 14, 13, 11, and six had a past history of recurrent UTI, diabetes, hypertension, and a Foley catheter of some type in place, respectively. Some patients had structural and or functional urinary tract dysfunctions such as renal stones, vesicoureteral reflux, and renal cysts. Seventy positive urine cultures were associated with UTI symptoms, while 16 cultures were associated with non-specific symptoms or symptoms not related to UTI, and four cultures were from asymptomatic individuals. The most common presentation was dysuria (40%), associated with other UTI symptoms such as hematuria, suprapubic pain, and frequency. Table 1 presents the patients’ characteristics and underlying medical conditions and symptoms at the time of positive urine culture for *S. aureus*.

**Table 1:** Characteristics of 85 patients and symptoms at the time of positive urine culture for Staphylococcus aureus.

Characteristics	Value
Mean age (years)	32.9
Median age (years)	28
Gender (percentage)	
Male	32 (38)
Female	53 (62)
Patient location at the time of ordering the urine culture (percentage)	
Inpatient	13 (15)
Outpatient	72 (85)
Underlying condition with the number of patients (percentage)	
Previously healthy	47 (52)
Diabetes	13 (15)
Hypertension	11 (13)
Foley’s catheter	6 (7)
Recurrent urinary tract infection	14 (17)
Renal stones	5 (6)
Vesicoureteral reflux	2 (2)
Renal cysts	2 (2)
Multiple bladder diverticulum	1 (1)
Neurogenic bladder	5 (6)
Benign prostatic hyperplasia	2 (2)
Single kidney	2 (2)
Symptoms (percentage)	
Fever	17 (19)
Hematuria	12 (13)
Suprapubic pain	16 (18)
Dysuria	36 (40)
Flank pain	22 (24)
Frequency	7 (8)
Urgency	5 (6)
Urine retention	2 (2)
Asymptomatic bacteriuria	4 (4)

The study included 77 patients for whom urinalysis was performed, of which 58 were positive for UTI. In Table 2, details about the frequency of positivity of the individual components of the urinalysis are provided. Twelve urinalyses were positive for nitrite, while 58 of them were positive for WBCs, 56 were positive for bacteria, and 38 were positive for RBCs.

**Table 2:** Urinalysis for 77 patients.

Result	No. of tests
Positive for nitrite	12
Positive for WBCs	58
Positive for RBCs	38
Positive for bacteria	56

**Figure 1:**
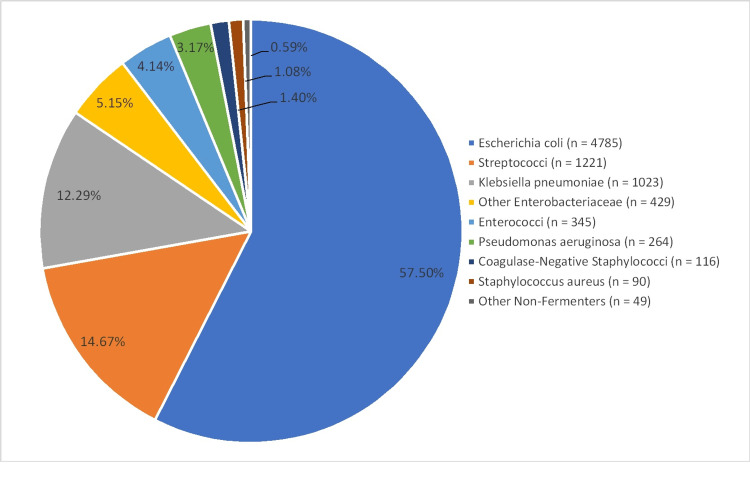
Percentage of S. aureus urinary tract infections among other uropathogens.

During the study period, we received 44,884 urine samples for culture and susceptibility testing. Among these urine cultures, 8322 (19%) were positive for uropathogens. *S. aureus* accounted for 1.08% of other uropathogens as presented in Figure 1.

Table 3 presents the susceptibility results of all 90 *S. aureus* isolates. Twenty-two (24%) of these were MRSA. All of these were susceptible to vancomycin, teicoplanin, and linezolid, and 91% were susceptible to trimethoprim/sulfamethoxazole.

**Table 3:** Number of susceptible isolates (%).

Antibiotic	No. of susceptible isolates (%)
Penicillin	12 (13%)
Oxacillin	68 (76%)
Erythromycin	77 (86%)
Clindamycin	84 (93%)
Gentamicin	87 (97%)
Trimethoprim-sulfamethoxazole	82 (91%)
Fusidic acid	61 (68%)
Tetracycline	78 (87%)
Vancomycin	90 (100%)
Teicoplanin	90 (100%)
Linezolid	90 (100%)

The antimicrobial resistance rate in MRSA isolates was much higher than methicillin-sensitive *S. aureus* (MSSA) UTI isolates, with P < 0.05, except for gentamicin and tetracycline. Table 4 shows the susceptibility results of MRSA and MSSA isolates.

**Table 4:** MSSA versus MRSA resistance rates (%) of the tested antibiotics. MSSA: methicillin-sensitive *Staphylococcus aureus*; MRSA: methicillin-resistant *Staphylococcus aureus.*

	MSSA	MRSA	P-value
Penicillin	56 (82)	22 (100)	0.034
Erythromycin	6 (9)	7 (32)	0.008
Clindamycin	2 (3)	4 (18)	0.013
Gentamicin	1 (1.5)	2 (9)	0.083
Trimethoprim-sulfamethoxazole (%)	1 (1.5)	7 (32)	0.000
Fusidic acid	14 (21)	12 (55)	0.008
Tetracycline	7 (10)	5 (23)	0.136

The MRSA-associated UTIs were significantly more prevalent in patients with underlying medical problems (P = 0.007).

While comparing outpatients with inpatients, we found that inpatients had higher percentages of elderly (38.5%), male (61.5%), and patients with underlying medical conditions and/or urinary structural abnormalities (53.8%). However, it was statistically insignificant with P > 0.05. See Table 5 for further details.

**Table 5:** Patients’ characteristics in relation to outpatient and inpatient settings.

	Outpatient	Inpatient	P-value
Age group			0.354
0-15 years	20 (27.8%)	4 (30.8%)	
15-45 years	36 (50%)	4 (30.8%)	
>45 years	16 (22.2%)	5 (38.5%)	
Gender			0.053
Male	24 (33.3%)	8 (61.5%)	
Female	48 (66.7%)	5 (38.5%)	
Underlying conditions			0.531
Previously healthy	40 (55.6%)	6 (46.2%)	
Patients with underlying medical conditions and/or urinary structural abnormalities	32 (44.4%)	7 (53.8%)	

## Discussion

*S. aureus* is not a frequent UTI pathogen in the general population. Correspondingly, in the present study, the prevalence rate of *S. aureus* bacteriuria accounted for only 1% of all urine isolates. Comparably, Goldstein conducted a laboratory-based study in France and found that *S. aureus* accounted for only 1.3% of isolates from urine specimens submitted from the community [9]. Similarly, in a multicenter community-based study conducted by Barrett et al. in Great Britain, *S. aureus* comprised only 0.5% of all UTI isolates [10].

It has been previously described that isolation of *S. aureus* from the urine is almost invariably associated with urine catheterization, or dissemination through a hematogenous route, affecting mainly elderly patients [4]. This fact is also supported by the research of Muder et al., who conducted a longitudinal study of 102 patients at a long-term veteran care facility with documented *S. aureus* bacteriuria and found that 13% of the patients with *S. aureus* isolated from their urine were bacteremic and 53% had bladder catheters [5]. The most common symptom associated with *S. aureus* UTI was fever. Other symptoms were hematuria, altered mental status, dysuria, suprapubic pain, and, less commonly, flank pain [5]. However, in our study, the picture was significantly different. The majority of the patients were female aged from 15 to 45 years. Interestingly, underlying medical conditions, urinary tract manipulations, or abnormalities were uncommon. Only 7% of the patients had indwelling urinary catheters. Blood cultures were not performed for most of the patients, possibly because the majority of the patients presented to the clinics with simple cystitis, and urosepsis was not initially suspected. The most common presentation was dysuria, which may represent simple cystitis rather than pyelonephritis. The possible explanation is our hospital setting where the majority of patients visit the emergency department and a variety of different specialty clinics. As many as 85% of the study subjects were outpatients. Therefore, our study mainly represented community-acquired infections.

A number of studies have indicated that the majority of *S. aureus* bacteriuria cases are not associated with symptoms of UTI and merely represent colonization. For example, Muder et al. found that only 33% of the patients with *S. aureus* isolated from their urine had UTI symptoms [5]. Likewise, in another retrospective cohort study, Capitano et al. identified 90 *S. aureus* infections in a single nursing home. Of these, 48% were classified as UTI using a definition of fever (temperature > 37.5°C) in association with staphylococcal bacteriuria, while 52% of the isolates were considered as just colonizers [6]. Furthermore, Pacio et al. reported that only 13% of long-term care patients colonized with MRSA at any site developed symptomatic UTIs [11]. In contrast, because of the different study population, 81.1% of the patients in the present study had UTI symptoms, and/or positive urinalysis for UTI, which represents true infection rather than colonization. Urinalysis is one of the most important tests to diagnose UTI and differentiate between colonization and true infection. However, it was not performed for eight patients in the present study, possibly due to typical symptoms of UTI, and only culture results were requested to determine the type of pathogen and susceptibility test results. The UTIs caused by MRSA are associated with longer stays in healthcare facilities, recent antibiotic use, and urinary catheterization [3,5]. In our study, out of 24 patients infected with MRSA, 16 patients had underlying medical conditions.

Several studies have reported different susceptibility patterns of *S. aureus* to a variety of tested antibiotics. This could be due to the selection criteria for *S. aureus* isolates and the study population. In a study of *S. aureus* biofilm formation in UTI, Yousefi et al. found that out of 39 *S. aureus* isolates, 30 isolates were MRSA. The overall susceptibility of *S. aureus* isolates to antimicrobial agents was 100% for linezolid and quinupristin/dalfopristin, 97.4% for chloramphenicol, 76.9% for trimethoprim-sulfamethoxazole, 64.1% for rifampin, 43.6% for clindamycin, 41% for nitrofurantoin, doxycycline, and gentamicin, 35.9% for erythromycin and ciprofloxacin, and 33.3% for tetracycline; however, vancomycin susceptibility was not reported in this study [2]. Yousefi et al. published another study about tigecycline- and vancomycin-resistant *S. aureus* strains among patients with UTI. The results revealed that the MRSA isolates were 100% susceptible to linezolid and quinupristin/dalfopristin but 93.3% were susceptible to vancomycin and tigecycline [12]. Antibiogram of *S. aureus* isolated from urine samples of patients with suspected cases of UTI performed by Akortha et al. showed the following results: the sensitivity pattern of *S. aureus* is 83%, 79.9%, 63.5%, 58.9%, and 50.2% to amoxicillin-clavulanic acid, ofloxacin, nitrofurantoin, amoxicillin, and gentamicin, respectively, while *S. aureus* isolates are 87.3%, 80.9%, and 79.3% resistant to trimethoprim-sulfamethoxazole, tetracycline, and nalidixic acid, respectively, but nothing is mentioned about vancomycin [13]. In our study, 24% of the isolates were MRSA. The antimicrobial susceptibility rate was much higher than the previously mentioned studies, which represents the susceptibility pattern of *S. aureus* causing UTI in the community. According to our results, 91% of *S. aureus* isolated from urine were susceptible to trimethoprim-sulfamethoxazole, which can be used as empiric treatment if *S. aureus* is suspected to be the cause of UTI or reported as a preliminary result before susceptibility results are available. Isolation of *S. aureus* from urine in patients without an obvious cause may indicate *S. aureus* bacteremia, therefore, it is necessary to perform blood cultures for any patient with positive *S. aureus* urine culture. As noted in the result section, resistance rates of the antibiotics in MRSA are much higher than MSSA UTIs, with a P-value < 0.05 for most of the antibiotics. This is understandable as some MRSA strains contain a plasmid that carries resistance genes for beta-lactams, fluoroquinolones, tetracycline, macrolides, clindamycin, and mupirocin [14].

## Conclusions

Although *S. aureus* UTI is known to be associated with other risk factors such as urinary catheterization, long hospital stay, or complicated UTI, our results show the community-acquired presentation of UTI. Trimethoprim-sulfamethoxazole may be used as an effective treatment for UTI caused by *S. aureus*. *S. aureus* UTI could be an alarming sign of more invasive infections such as *S. aureus* bacteremia, though clinical evaluation and finding the source of *S. aureus* are crucial for effective treatment and prevention of further complications.
